# Characterization and assessment of the genotoxicity and biocompatibility of poly (hydroxybutyrate) and norbixin membranes ^1^

**DOI:** 10.1590/s0102-865020200070000006

**Published:** 2020-08-28

**Authors:** Rayssilane Cardoso de Sousa, Luiz Fernando Meneses Carvalho, Antônio Luiz Martins Maia, Danniel Cabral Leão Ferreira, Fabrício Pires Moura do Amaral, Lianna Martha Soares Mendes, Vicente Galber Freitas Viana

**Affiliations:** I Fellow PhD degree, Postgraduate Program in Biotecnology, Rede Nordeste de Biotecnologia (RENORBIO), Universidade Federal do Piauí (UFPI). MSc, Materials Engineering, Instituto Federal de Educação, Ciências e Tecnologia (IFPI), Teresina-PI, Brazil. Conception and design of the study, technical procedures, acquisition and interpretation of data, technical procedures, manuscript preparation.; Instituto Federal de Educação, Ciências e Tecnologia, Teresina, PI, Brazil; II PhD, Full Professor, Postgraduate Program in Material Engineering, IFPI, Teresina-PI, Brazil. Technical procedures.; III PhD, Biomedical Engineering, Universidade do Vale do Paraíba (UNIVAP), Sao Jose dos Campos-SP, Brazil. Associate Professor, Health Sciences Center (FACIME), Universidade Estadual do Piauí (UESPI), Teresina-PI, Brazil. Technical procedures, microscopic examinations, interpretation of data.; Health Sciences Center, Universidade Estadual do Piauí, Teresina, PI, Brazil; IV Fellow Master degree, Professional Master’s Program in Human and Animal Biotechnology MPBiotec, UESPI, Teresina-PI, Brazil. Technical procedures.; V PhD in Pharmacology, Universidade Federal do Ceará (UFC), Fortaleza-CE, Brazil. Associate Professor, FACIME, UESPI, Teresina-PI, Brazil. Technical procedures, microscopic examinations, interpretation of data.; FACIME, UESPI, Teresina, PI, Brazil; VI PhD in Medical Sciences, Faculdade de Medicina, Universidade de Brasília (UnB). Associate Professor, Health Sciences Center (CCS), UFPI, Teresina-PI, Brazil. Histopathological examinations, interpretation of data.; Health Sciences Center, UFPI, Teresina, PI, Brazil; VII PhD in Sciences (Applied Physics), Instituto de Física de São Carlos, Universidade de São Paulo (USP). Associate Professor, Postgraduate Program in Material Engineering, IFPI, Teresina-PI, Brazil. Scientific and intellectual content of the study, technical procedures, critical revision, final approval.; Postgraduate Program in Material Engineering, IFPI, Teresina, PI, Brazil

**Keywords:** Biocompatible Materials, Hydroxybutyrates, Genotoxicity, Wound Healing, Rats

## Abstract

**Purpose:**

To synthesize and characterize poly(hydroxybutyrate) (PHB) and norbixin membranes to evaluate them for genotoxicity in rats and wound healing in mice by histological staining.

**Methods:**

For the evaluation of genotoxicity, male rats ( *Rattus novegicus* ) were divided into three groups (n= 5): 5% PHB/Norbixin membrane introduced into the peritoneum by laparotomy; B – negative control; C – positive control (intraperitoneal dose of cyclophosphamide 50 mg/kg). For the evaluation of biocompatibilty, a cutaneous wound was induced on the back of males mice ( *Mus musculus* ) divided into two experimental treatment groups: control and membrane that underwent euthanasia after 7 and 14 days treatment. Statistical analysis ware made by One Way Anova post hoc Tukey Test (p<0.05).

**Results:**

Regarding the incidence of polychromatic erythrocytes, there was no difference between negative control and 5% PHB/Norbixin membrane; however, when compared to the positive control represented by cyclophosphamide, there was a significant difference (p <0.001). As for DNA damage, the changes induced in the first 4h were repaired in 24h. In addition, the membrane was effective in abbreviating the inflammatory process and served as a scaffold due to the stimulus to reepithelialization mainly on the 7 days of treatment.

**Conclusion:**

The non-genotoxic PHB/Norbixin 5% membrane presented promising results that suggest its effectiveness as a guide for tissue regeneration given its biocompatibility.

## Introduction

Biomaterials are devices that are in contact with biological systems, for diagnostic, vaccinal, surgical or therapeutic applications, and are made of compounds of natural or synthetic origin, as well as of chemically modified natural materials; a great variety of these products have been used in health care^1 , 2^ .

There are increasing studies involving the use of biomaterials in the field of tissue engineering for healing purposes, in which biocompatible, biofunctionality and non-toxic scaffolds can interact with tissues and stimulate total or partial reconstitution^1 , 3 - 5^ .

In addition to the ability to replace damaged tissue, biomaterials must be capable of providing adequate mechanical support to the patient, as well as of being biocompatible with the tissue and of presenting low rejection of the implant by the organism, such as in the constitution of artificial organs and wound dressings, wound healing, bone repair and fractures, implantable materials (sutures, plaques, heart valves, teeth), hemodialysis systems, devices for the controlled release of drugs, biosensors, blood circulation tubes^6 - 9^ .

Non-synthetic biopolymers such as polyhydroxyalkanoates, which are polymers synthesized by bacteria in the presence of renewable carbon sources, such as sugarcane, are matrix options for biomaterials, among which the polyhydroxybutyrate, C_4_H_6_O_2,_ can be used as a biodegradable polymer, with high potential for biocompatibility and bioreabsorption and, when implanted in living systems, do not require subsequent removal of the body and do not cause undesirable effects in the long term^10 - 13^ .

Polymeric matrices can be associated to several constituents in the form of composites aiming at the aggregation of important properties that can optimize their application in biological environment.

Norbixin is a carotenoid pigment extracted from the annatto seed pericarp ( *Bixa orellana* ). The annato tree is native to Central and South America. Carotenoids have antioxidant properties and act against free radicals, which biologically favors the evolution of the tissue repair process. Animal studies have revealed the absence of genotoxic, teratogenic or mutagenic effects from these materials^14 - 17^ .

The use of polymer matrices for wound healing, as well as natural extracts, such as poly (hydroxybutyrate) and norbixin, can be promising, although there are no experimental studies involving norbixin associated with polyhydroxybutyrate as constituents of biomaterial in the repair of cutaneous wounds or bone regeneration^9^ .

Moreover, Sousa *et al* .^18^ synthesized a membrane from poly (hydroxybutyrate) (PHB), norbixin and ethylene glycol and concluded that the micronucleus test suggested that the biomaterial has no biological toxicity as it does not change the incidence of polychromatic erythrocytes. The comet assay revealed an increase in DNA damage in the first 4h, suggested a repair of the same in 24 hours.

The synthesis of a membrane from a non-synthetic biopolymer (PHB) and a natural antioxidant (norbixin) as a healing biomaterial from renewable resources requires both physico-chemical characterization and biocompatibility tests. The objective of this study was to synthesize and evaluate the physicochemical properties of poly (hydroxybutyrate) membranes containing or not norbixin ( *Bixa orellana* L.) to assess its genotoxic potential in rats and wound healing in mice in histological staining.

## Methods

The present study was approved by the Animal Research Ethics Committee (CEUA), Universidade Estadual do Piauí (UESPI) under protocol number 09241/2016, in accordance with the Guiding Principles for the Care and Use of Laboratory Animals.

### Materials

The norbixin used in this study was extracted from the seeds of Annato ( *Bixa orellana L* .), collected in the city of Teresina-PI, whose registration was carried out at the Herbarium Graziela Barroso -TEPB, at the Federal University of Piauí (nº 31573). The polymer used as matrix of the membranes was PHB, which was supplied by the company PHB Industrial S/A (Serrana, SP - Brazil) and was prepared from the fermentation of sucrose by bacteria “ *Alcaligenes eutrophus* ”. Potassium hydroxide (KOH), ethyl ether, hydrochloric acid (HCl) and chloroform (Synth) all P.A.

Norbixin was extracted from annatto seeds ( *Bixa orellana* L.). Initially, seeds were mixtured with ethyl ether under stirring (to the supernatant of the seeds formed from that reaction, was added KOH solution 4%, heating at 70°C). After cooling the mixture, HCl was added until a color change precipitate was formed. The precipitate was washed until pH was approximately equal to 4 and then oven dried at 70°C for 24 hours.

As for the preparation of the membranes, three types of membranes were prepared and in each of them the mass of PHB was kept constant. Membrane 1 was synthesized only with PHB. Membrane 2 was prepared with norbixin content of 5% of the mass of PHB. Membrane 3 was prepared with norbixin content of 10% of the mass of PHB.

Preparation of membranes: (a) initially the PHB was allowed to rest in chloroform, (b) the PHB held in chloroform was heated to 60°C under stirring. (c) In the preparation of the membrane containing only PHB, after the solution reached room temperature, the mixture remained for 10 min on ultrasound and finally was then poured onto petri dish and allowed to stand for 24 hours. On the 5% PHB/norbixin membrane, as well as on the 10% PHB/norbixin membrane, after step (b), the norbixin pigment previously diluted in chloroform was added to the PHB following the same final process as step (c). The thickness of the membranes was measured using a 0mm-12.7mm digital Micrometer Western Tool^®^ (California, EUA).

### Membrane characterization techniques

**Fourier Transform Infrared Spectroscopy (FTIR)** *.* The membrane spectra were obtained in infrared IRAffinity-1 Shimadzu (Kyoto, JAPAN) with the recording range of waves from 4000 to 400 cm^-1^. The spectra were performed at a resolution of 16 cm^-1^ from 45 scans.

**Thermogravimetric Analysis (TA) and Differential Scanning Calorimetry (DSC)** . The thermogravimetric analysis of each sample was performed by using an average mass of 8.0 mg. The analyses were obtained in the temperature range of 30 to 900°C, at a heating rate of 10° C.min^-1^ under a nitrogen atmosphere, with gas flow of the order of 50mL.min^-1^. All results were obtained with tests performed on the TGA-51 Shimadzu (Kyoto, JAPAN). Differential Exploration Calorimetry was performed in a DSC-60 Plus Shimadzu (Kyoto, JAPAN), under the same TGA conditions, with an average mass of 6 mg at a temperature ranging from 30 to 500°C.

**Scanning Electron Microscopy (SEM)** . The samples were initially metallized with gold. The images were obtained by using the scanning electron microscope SURPERCAN SSX-550 Shimadzu (Kyoto, JAPAN), to a 15.0 kV voltage, magnification at 2,000x.

### Genotoxicity assessment

The same group of samples composed of 15 *Rattus novergicus* male animals with average weight between 200 and 300g were used simultaneously in both tests. The animals were divided into three groups (A - negative control, B - exposed to PHB/Norbixin 5% membrane and C - positive control) as each intervention was performed. The 5% PHB/Norbixin membrane was chosen to perform the experimental procedures because it presented intermediate characteristics between both pure PHB membranes and PHB/Norbixon 10% membranes.

In group A, there was no treatment, only a peritoneal laparotomy was performed to simulate the surgical process. In group B, which was exposed to biomaterial, a laparotomy was performed for implantation of the membrane in the peritoneum region. In group C, as a single dose injected of intraperitoneal cyclophosphamide (genotoxic and inducing DNA damage of bone marrow cells) at a concentration of 50 mg/kg per animal. Each experimental group was composed of 05 animals anesthetized with ketamine (1.0 ml/kg) and xylazine (1.1 ml/kg) intramuscularly. The animals were sacrificed 72h after the experiment had started.

**Micronucleus test** . The micronucleus test was carried out by using bone marrow cells collected immediately after each animal was sacrificed. A 1.0 ml syringe filled with fetal bovine serum was introduced into the opening at one end of the femur. The bone marrow material in the fetal bovine serum was resuspended till it got homogeneous. The suspension was centrifuged for 5 minutes at 1000 rpm, and the supernatant was discarded at the end of the procedure by a Pasteur Pipette. Two slides per animal were made.

Cells were re-suspended, placed on slides and stained with 10% GIEMSA in phosphate buffer (pH 6.8, for 10 min). Then, the cells were observed under an optical microscope (100X), in a short time in a NIKON LABPHOT 2 microscope (New York, EUA)^19 - 22^ .

**The comet test** *.* For the comet assay, blood samples were collected from the animal’s tail in two different occasions: 4h post-exposure (acute) and 24h post-exposure (chronic) to the biomaterial. At the end of each exposure (4h or 24h), 40 μL of the blood samples collected from the animal’s tail were transferred to microtubes containing 120 mL of low melting point agarose (1.5%) at 37°C. This mixture was homogenized and transferred to precoated slides with 5% agarose. The slides were then covered with coverslips and placed at 40°C for 30 minutes. The coverslips were removed and the slides were immersed in lysis solution (NaCl (2.5 M), EDTA (100 mM) and 1.2 g TRIS (10 mM), 1% Triton X-100 and 10% DMSO].

For DNA denaturation, the slides were placed in an electrophoresis cell containing a buffer solution pH> 13 (300 mM NaOH and 1 mM EDTA), prepared from a stock solution (NaOH and 200 mM EDTA, pH 10.0) for 20 min. The electrophoresis was performed at 25 V and 300 mA at a temperature of 4°C for 15 minutes. The slides were then immersed in a neutralization solution (0.4M Tris, pH 7.5, 3 cycles of 5 minutes each).

The analysis was performed on an immunofluorescence by Biofocus Stereomicroscope WF10X/22 (x40) (California, EUA) equipped with a 420-490 nm filter and a 520 nm barrier filter. The images were captured with a 5.0 megapixel CCD digital camera for immunofluorescence. DNA damage was assessed by measuring the percentage of DNA in the tail and by the height/length of the tail, which were calculated at 100 nucleoids/ sample (two slides per animal). In order to get that, the OpenComet software was used^23^ .

### Wound healing assessment

In this study, 20 male *Mus musculus* mice, with a mean weight ranging from 20 to 30g, were randomly divided into two groups: control and membrane (n= 10 animals each). Five animals of each group were sacrificed after 7 days and the remaining five mices after 14 days of treatment.

**Induction of experimental injury** *.* The animals were weighed and then anesthetized by intramuscular administration of 10% ketamine hydrochloride at a dose of 0.1 ml for each 100g of body weight associated with the same dose of 2% xylazine hydrochloride. Anesthetic drugs were applied dissociatively, using 1 mL capacity syringes and 8 x 0.30 mm needles. The lesion was induced on the back of the animals, positioned in dorsal decubitus, and began with the tricotomy of the region. To perform the experimental lesion, a surgical instrument (punch) 8 mm in diameter, positioned perpendicular to the back was used. In group membrane, the PHB/Norbixin 5% membrane was implanted.

**Histological analysis** *.* The mice were submitted to euthanasia by an overdose of sodium thiopental in the concentration of 50 mg/kl. The surgical specimen was removed and after fixation for 24h, the histological technique of routine was processed, including the steps of gradual dehydration, diaphanization, infiltration and embedment in paraffin. The samples were submitted to longitudinal histological cuts, stained with hematoxylin eosin (HE) and analyzed by microscope Olympus CX31 (Tokyo, JAPAN), with x400 magnification. A qualitative histological analysis of inflammatory reaction was made, defined by: reepithelization, granulation tissue, presence of inflammatory cells, fibroblasts, collagen deposition and neovascularization. The criteria of histological analysis followed the protocol of Meirelles *et al* .^24^ to facilitate statistical analysis, the following score was created: 0, 1, 2 and 3, where: 0 –absent, 1 – discrete, 2 – moderate, 3 – intense.

### Statistical analysis

Data were analyzed using One-Way ANOVA test post hoc Tukey. For all tests was considered a 5% significance level. The results were expressed as mean, or standard deviation of three independent experiments. The result was considered positive if there was a statistically significant increase (p <0.05).

## Results

Norbixin and PHB have good solubility and processability in chloroform, facilitating the formation of membranes. They presented good consistency with an averge thickness of approximately 400 μm, apparently homogeneous.

### Physical chemical characterization

**Figure 1:**
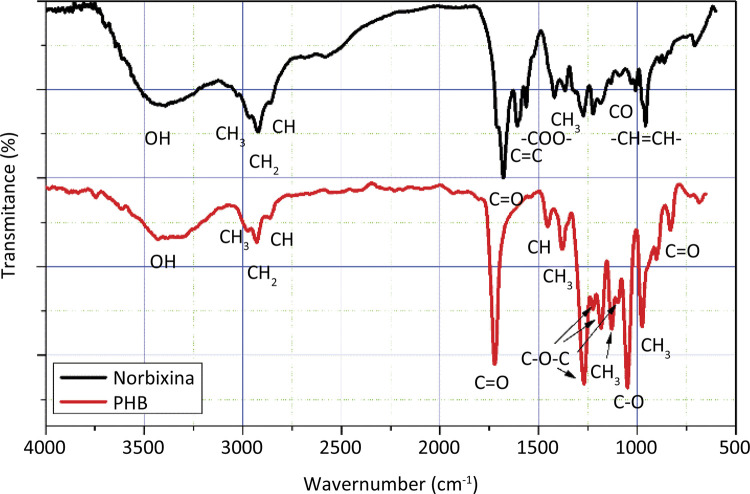
- Infrared spectra of norbixin and PHB membrane.

The infrared spectra of norbixin and polyhydroxybutyrate can be seen in Figure 1 . Norbixin has a band in the region of 3762-3080 cm^-1^, regarding the OH groups of its two terminal carboxylic acids. The 2921 cm^- 1^ peak, which has two shoulders at 2985 and 2921 cm^-1^, is a stretching of the CH_2_ group, with the shoulders representing symmetrical stretches of both the CH_3_ and CH groups, respectively. Peaks at 1678, 1563, 1134, 1091, 1034 cm^- 1^ , represent the terminal carbonyls found in the norbixin structure. The peak at 1611 cm^-1^, and the peaks in the region between 1000-886 cm^-1^ are attributed to the double bonds. The methyl groups present in the norbixin chain are represented by the peaks at 1422 and 1364 cm^-1 25 - 27^ .

The PHB also has a symmetrical stretching peak of the CH_2_ group at 2930 cm^-1^ with two shoulders with spectra at 2977 and 2861 cm^-1^, depicted the CH3 and CH groups, respectively. Peaks at 1722, 1269, 1224, 1183, 1098, 1049 and 830 cm^-1^ represent bonds of carbon atoms to oxygen atoms (CO groups). The methyl (CH_3_) groups show peaks at 1380, 1129 and 974 cm^-1 28^ .

**Figure 2:**
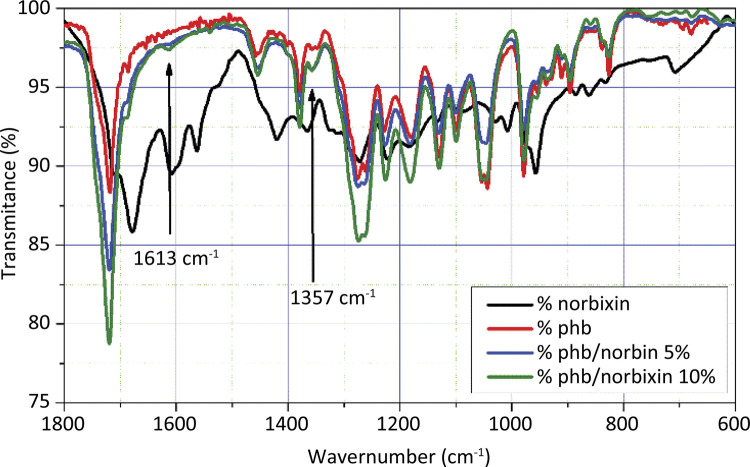
FTIR spectroscopy of norbixin, PHB and PHB/Norbixin membranes (5 and 10%).

The spectra of both PHB/Norbixin 5% and 10% membranes compared to the PHB membrane have not showed major modifications concerning the formation and disappearance of PHB peaks ( Fig. 2 ).

**Figure 3:**
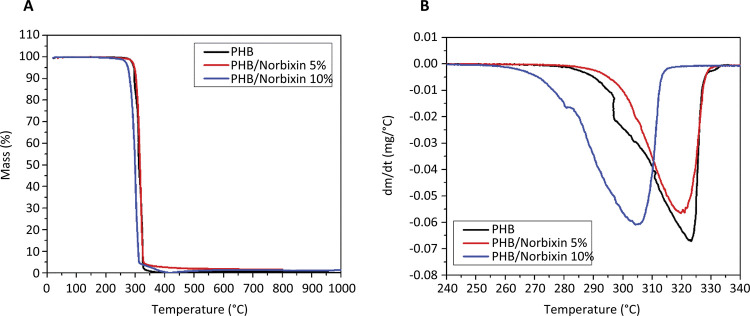
Thermogravimetry of norbixin samples and PHB membranes: a) thermogravimetric curve and b) derivative thermogravimetric curve (DTG).

In Figure 3a and b , PHB membranes degrade with a percentage below 0.5% between temperatures 25 to 250°C, whereas norbixin loses approximately 2.0% mass between 25 and 150°C, which would probably be water of constitution^29^ .

**Figure 4:**
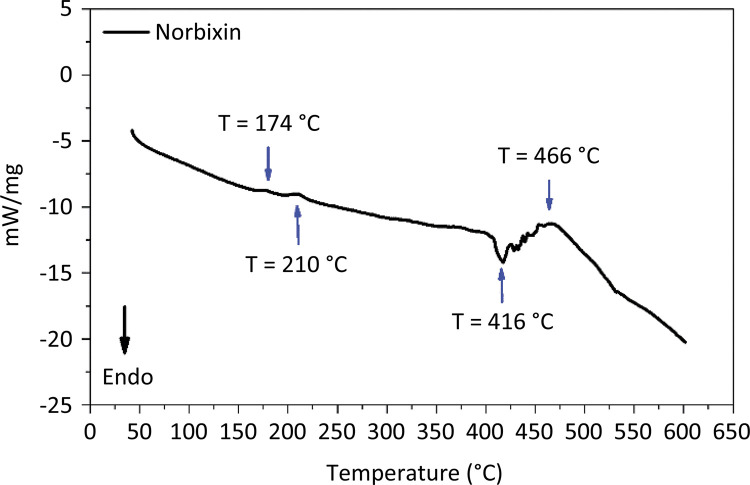
DSC curve of norbixin.

In the DSC curve of norbixin, four characteristic thermal processes are observed. Three of them are of exothermic origin - at temperatures of 174, 210 and 466°C ( Fig. 4 ). The fourth process is an endothermic one and occurs at 416°C, which is similar to a phase change process, and may, therefore, represent a process of melting or boiling of one of the components of norbixin degradation. The norbixin DSC curve did not show any event associated with a phase change process up to that temperature.

**Figure 5:**
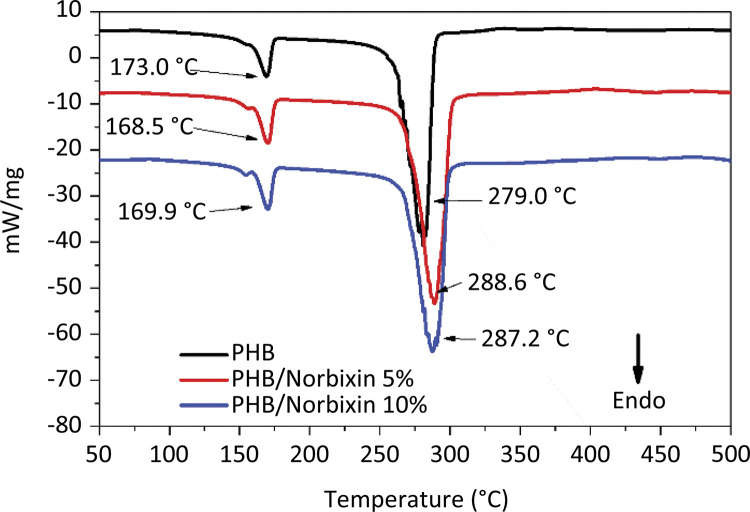
Differential scanning calorimetry of the samples.

The DSC curves of the PHB membranes show endodermal processes, with distinct crystalline behavior of PHB, which is similar to other studies^30^ ( Fig. 5 ). They are well-defined peaks which indicate that the PHB used has an excellent degree of purification. The first endodermal event observed on each of the membranes occurs at 169°C (PHB membrane) and at 170°C on 5 and 10% PHB/norbixin membranes. According to Machado *et al* .^31^ this process represents the merger of PHB. The addition of norbixin to the membranes does not cause considerable variation in this phase transition process.

Regarding the second endothermic event, a clear change in the temperature of this process is observed, showing that the increase in norbixin content has a considerable effect on itself. In the PHB membrane, this process occurs at 280°C: on the 5% PHB/norbixin membrane at 287°C; on the 10% PHB/norbixin membrane at 289°C. In Table 1 , it can be observed that the energy involved in the second event oconcerning all the membranes is much larger than the one occurring in the first one. That result may indicate that the second event would not merely be a boiling phase transition once it occurs in the degradation range seen on the TG curve of the PHB membranes.

**Table 1:** - DSC Score of the Samples.

Thermic Processes	PHB	PHB/Norbixin 5%	PHB/Norbixin 10%
Melting Point, MP (°C)	170.0	169.0	169.0
Heat Flow, HF (mJ)	316.2	423.3	390.0
“Boling Point”, BP (°C)	280.0	287.0	289.0
Heat Flow, HF (mJ)	3.4 x 10^3^	5.0 x 10^3^	4.5 x 10^3^

**Figure 6:**
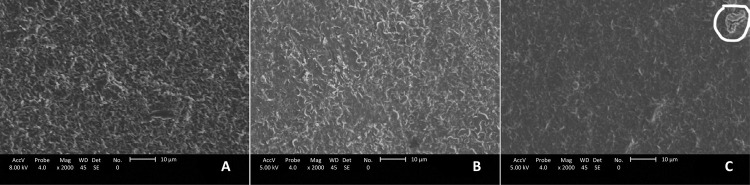
- a) PHB membrane, b) 5% PHB/norbixin membrane, and c) 10% PHB/norbixin membrane (x2.000 Magnification scanning electron microscopy).

In Figure 6 , the micrographs of the samples, magnification at 2000x by SEM are observed. The Figure 6c presented fewer clusters, suggesting greater aggregation and dissolution of norbixin in the PHB matrix.

### Genoxicity assessment

The 5% PHB/Norbixin membrane was chosen to perform the experimental genotoxicity procedures once it presents intermediate characteristics between PHB and 10% PHB/Norbixin membranes observed in the physical-chemical characterization.

**Figure 7:**
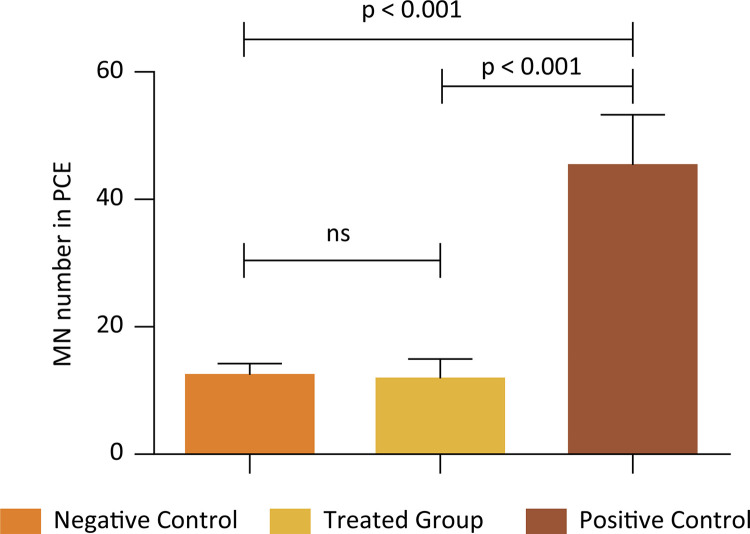
Mean of Micronuclei found in 2000 polychromatic erythrocytes after a 72h-exposure.

Both Table 2 and Figure 7 show the absolute frequency of PCEMNs and the mean values of micronuclei along with their representative percentage in the analyzed groups, as well as the mean and standard deviation of these results.

**Table 2:** Frequency of micronucleated polychromatic erythrocytes (PCEMNs) in bone marrow *Rattus norvegicus* exposed to PHB/Norbixin membrane after 72h.

Treatment	Number of PCES Analyzed	PCEMNs		Mean ± Standard Deviation

		Nº	%	
Distilled Water	10.000	61	0.0061	12.2 ± 1.92
Membrane	10.000	60	0.0060	12.0 ± 2.95
CPA (50mg/Kg)	10.000	226	0.0226	45.2 ± 8.25

Regarding the membrane exposure process, after 72h, there was no significant increase in the genotoxicity checked by the micronucleus test when compared to the negative control, since there was no statistical change in the incidence of polychromatic erythrocytes in rats (p> 0.05). When compared to the positive control represented by cyclophosphamide, known to be genotoxic, there was a significant difference (p <0.001).

Concerning the evaluation of genotoxicity by the comet assay in the group exposed to the 5% PHB/Norbixina membrane, the results indicated an increase in damage in the first 4h of exposure evaluated by the percentage of DNA in the tail ( Table 3 ). This result significantly regressed after 24h of exposure. Therefore, the genotoxic effect was apparently repaired after this period. This observation is compatible with the other analyzed parameters, such as TailMoment and tail length.

**Table 3:** - DNA damage found per group after exposure to 5% PHB/Norbixin membrane.

Paramters	Positive Control	Negative Control	Membrane 4h	Membrane 24h
% DNA tail	6.8 ± 1.1	1.1 ± 0.2	4.4 ± 0.98	1.1 ± 0.34
Tail length (μM)	17.0 ± 2.0	7.6 ± 2.1	11.3 ± 1.23	5.9 ± 0.88
TailMoment	1.9 ± 0.1	0.8 ± 0.6	1.0 ± 0.28	0.5 ± 0.19

### Wound healing assessment

**Figure 8:**
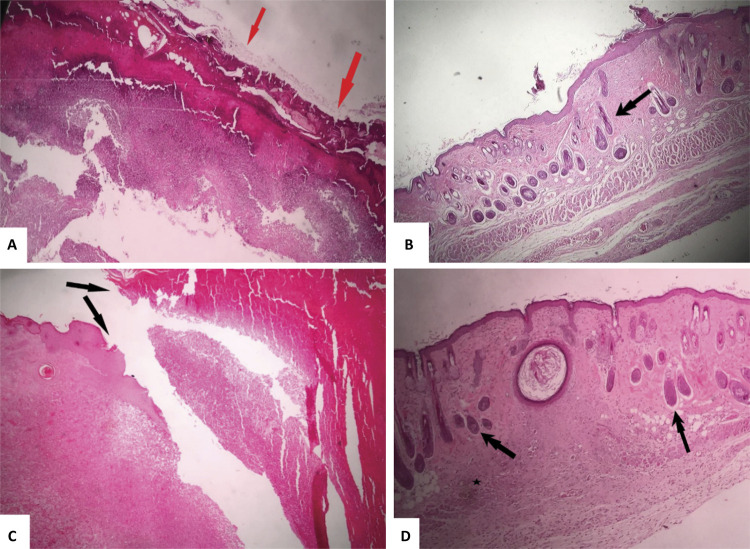
Illustrative photograph of the microscopic analysis of the different groups using a x4 objective: a) Control and keratin in the surface of the epidermis, indicated by the red arrows at 7 days. b) Membrane (presence of hair follicles, double arrows) at 7 days. c) Control (Inflammatory infiltrate ( *asteristic* ) and ulceration ( *black arrow* ) at 14 days and d) Membrane at 14 days.

The different treatment groups in both observation periods of 7 and 14 days are shown in Figure 8 . The histological quality and speed of the cicatricial reorganization of the groups treated with Norbixin/PHB 5% membrane compared to the other groups at 7 and 14 days is notorious, the results of which were also observed in Table 4 of quantitative histological evaluation.

**Table 4:** Summary of the quantitative results of the histological analysis (%) in the observation period of 7 and 14 days.

Criteria	Treatment groups (score)	

	Control	Membrane
	7 days	
Edema	2	1
Acute inflammation	3	1
Chronic inflammation	2	1
Mixed inflammation	2	1
Granulation tissue	2	2
Neovascularization	2	2
Presence of fibroblasts	2	2
Necrosis	2	0
Reepithetization	0	3
	14 days	
Edema	2	2
Acute inflammation	2	3
Chronic inflammation	2	2
Mixed inflammation	2	2
Granulation tissue	2	2
Neovascularization	2	2
Presence of fibroblasts	2	2
Necrosis	1	2
Reepithetization	2	2

## Discussion

### Physical chemical characterization

With the addiiton of norbixin to the membranes, a peak at 1613 cm^-1^ comes up, probably representing the double bonds of norbixin. At 1357 cm^-1^ one has the definition of a peak, which apparently in the PHB is represented by two small peaks at 1357 and 1347 cm^-1^. This norbixin peak represents the methyl groups attached laterally to its backbone. It has been found that the addition of 5 and 10% norbixin to the PHB membrane changes the peak intensity at 1722, 1453, 1380, 1269, 1224, 1183, 1129, 1098, 1049, 974 cm^-1^, suggesting good interaction between the componenets of the membrane.

The degradation of both PHB and PHB/Norbixin membranes occurs almost entirely in a single stage, between 243 and 272°C. This result is in accordance with the results found in the literature for polyhydroxybutyrate^27^ . In this region, PHB has a degradation rate of 99.1%; the 5% PHB/Norbixin membrane, 96.9%; and the PHB/ Norbixin 10%, has a 96.3% loss of mass. It is observed that as the norbixin concentration increases in the membranes, the initial temperature of the main degradation decreases, going from 269°C to 243°C.

Norbixin, unlike PHB, has degraded in more than one stage. The first degradation occurred from 25 to 120°C, a loss of 2.0% in its mass probably represented by the release of water. The second degradation band, with approximately 45.2% mass loss, occurred during the range from 145 to 440°C. A slow third degradation, initiated at 440°C, lasts until a temperature above 1000°C is reached. At 1000°C a residual mass of approximately 8.0% remains. Similarly, for to Scootter^32 , 33^ , the norbixin degradation itself starts at 130°C.

This result has also been reported in the study of Reith and Gielen^34^ who observed that norbixin does not melt up to a temperature of 300°C. These authors observed that norbixin changes color from 250°C, changing gradually from red color to a darker color. This fact was also reported by Scotter *et al* .^33^ who stated that norbixin undergoes carbonization above 290°C, changing from red to a dark color. Probably the endothermic processes found on the norbixin DSC curve are also associated with the decomposition of norbixin.

Regarding the TG and DSC thermal measurements, a thermal behavior of gradual mass decomposition of the membranes was observed in a single stage, which is a typical behavior of PHB. The displacement of the curves to the right of the PHB/Norbixin membranes in relation to the PHB ( Fig. 5 ) is associated to the fact that the norbixin degradation (from 176°C on) occurs in several stages and at temperatures lower than the the PHB temperatures, suggesting and colligative interactions between both structures due to the lower degradation temperature of the PHB/Norbixin membranes in relation to the pure PHB membrane - an interesting property for its application as biomaterial. These physico-chemical interactions were reported in other studies and were also seen in the FTIR, given the decrease in peak intensity^25 , 28 , 31^ .

The membranes presented a homogeneous, non-porous surface, which revealed characteristic spectra of their functional groups and that reflected the adhesion and aggregation of these two components by MEV. The clusters were observed in the Figure 6 are characteristic of the PHB^31^ .

### Genoxicity assessment

Based on the mentioned results, the micronucleus test suggested that the biomaterial has no biological toxicity inasmuch as the incidence of polychromatic erythrocytes in the blood of *Rattus novergicus* has not changed.

Although the results of the comet assay have shown an increase in the DNA damage within the first 4h, they suggested repairing such damage at 24h using the membrane. The results were promising when it comes to the use of norbixin and PHB as biomaterials, though there are few studies in literature that have evaluated both biological effects and properties of norbixin in animal tissues.

The in vivo assays used in this research were relevant because they show detailed biological and physiological information and are officially approved and considered as by the Organization for Economic Cooperation and Development (OECD) as part of the security prootcol analysis for genotoxicity assessment^35 , 36^ .

### Wound healing assessment

In this study, biomaterial used as scaffold in the repair of cutaneous wounds in mice was effective in both experimental periods, with complete cicatrization of the induced lesion, suggesting the biocompatibility of the biomaterial in the biological system in question. However, it was observed that such scaffold was more effective in the acute phase, that is, in the first 7 days of the lesion after the experiment with the presence of mild inflammatory infiltrate, than at 14 days, because there was a moderate and more intense inflammatory infiltrate. Considering that the literature reports that, in intact tissue, carotenoid pigments are protected from oxidation, the increase of the inflammatory process could be related to the decomposition of norbixin during the observational period of the experiment^37 - 40^ . Especially in the first days of tissue injury, it was observed that the PHB/Norbixin 5% membrane presented promising results that suggest its effectiveness as a guide for tissue regeneration and abbreviation of acute inflammation.

Alves *et al* .^41^ evaluated the therapeutic effect of biomaterial with polymer matrix associated with norbixin compared to laser photobiomodulation (λ = 780 nm, ED = 6 J/cm^2^, P = 60 mW, t = 4s, postoperatively on alternate days until they were euthanized), which was associated with or without a polystyrene membrane coated with norbixin and collagen (PSNC) on bone healing in rats with calvarial bone defect. The groups were analyzed after 15 and 30 days treatment. The authors concluded that the PSNC membrane was effective in reducing the inflammatory process and served as a scaffold for bone repair. The laser PBM only showed positive effects on the bone repair process with increased deposition and organization of the newly formed bone. However, the association of laser PBM + membrane failed to improve the bioactive properties of the membrane scaffold.

Use of biomaterials with polymeric matrices in the health field^42^ is known in the literature and the promising efficacy of the biomembrane as a guide for tissue regeneration was expected. However, studies that investigated the use of norbixin such as biomaterials are recent^13 , 15 , 16 , 18^ , since the most common reports still surround their application in the food industry. It is necessary to continue this research line considering the potential for application of the formulated biomaterial, as observed in this study mainly in the first days after tissue injury, necessitating the continuity of this pilot study with longer intervals of observation time, larger samples and increased inflammatory analyses, immunohistochemistry and biochemistry to know the physico-chemical interactions of this biomaterial in an isolated biological system.

## Conclusions

Homogeneous, non-porous membranes were obtained, which revealed characteristic spectra of their functional groups and that reflected the adhesion and aggregation of these two components by MEV. In addition, the decrease in temperature was observed as the norbixin content increased, which may optimize its use as biomaterial, since it suggests a better degradation of the PHB/Norbixin membrane regarding the PHB membrane. The micronucleus test and comet assay presented results that corroborate with the 5% PHB/Norbixin membrane suitability for biological purposes and the polyhidroxybutyrate membrane coated with norbixin reduced the inflammatory process and served as a scaffold for wound healing when used in isolation.

## Footnotes

1 Research performed at Laboratory of Postgraduate Program in Materials Engineering (PPGEM), Instituto Federal de Educação, Ciências e Tecnologia (IFPI), and Núcleo de Biotecnologia e Biodiversidade, Universidade do Estado do Piauí (UESPI), Teresina-PI, Brazil. Part of the Master degree thesis, Postgraduate Program in Materials Engineering, IFPI. Tutor: Prof. Dr. Vicente Galber Freitas Viana.
